# NLR-Dependent Regulation of Inflammation in Multiple Sclerosis

**DOI:** 10.3389/fimmu.2017.02012

**Published:** 2018-01-18

**Authors:** Marjan Gharagozloo, Katsiaryna V. Gris, Tara Mahvelati, Abdelaziz Amrani, John R. Lukens, Denis Gris

**Affiliations:** ^1^Program of Immunology, Faculty of Medicine and Health Sciences, Department of Pediatrics, CR-CHUS, University of Sherbrooke, Sherbrooke, QC, Canada; ^2^Center for Brain Immunology and Glia, Department of Neuroscience, School of Medicine, University of Virginia, Charlottesville, VA, United States

**Keywords:** NLRs, multiple sclerosis, NLRP3, NLRP12, NLRX1, inflammation, astrocytes, microglia

## Abstract

Multiple sclerosis (MS) is an autoimmune disease of the central nervous system (CNS) associated with inappropriate activation of lymphocytes, hyperinflammatory responses, demyelination, and neuronal damage. In the past decade, a number of biological immunomodulators have been developed that suppress the peripheral immune responses and slow down the progression of the disease. However, once the inflammation of the CNS has commenced, it can cause serious permanent neuronal damage. Therefore, there is a need for developing novel therapeutic approaches that control and regulate inflammatory responses within the CNS. Nucleotide-binding oligomerization domain (NOD)-like receptors (NLRs) are intracellular regulators of inflammation expressed by many cell types within the CNS. They redirect multiple signaling pathways initiated by pathogens and molecules released by injured tissues. NLR family members include positive regulators of inflammation, such as NLRP3 and NLRC4 and anti-inflammatory NLRs, such as NLRX1 and NLRP12. They exert immunomodulatory effect at the level of peripheral immune responses, including antigen recognition and lymphocyte activation and differentiation. Also, NLRs regulate tissue inflammatory responses. Understanding the molecular mechanisms that are placed at the crossroad of innate and adaptive immune responses, such as NLR-dependent pathways, could lead to the discovery of new therapeutic targets. In this review, we provide a summary of the role of NLRs in the pathogenesis of MS. We also summarize how anti-inflammatory NLRs regulate the immune response within the CNS. Finally, we speculate the therapeutic potential of targeting NLRs in MS.

## Introduction

Inflammation is a key component that accompanies the pathophysiology of all diseases (1). Multiple sclerosis (MS) is a neurodegenerative and demyelinating disease with a well-defined inflammatory component. Therefore, homeostatic processes that regulate inflammation may yield important insights into pathophysiology of MS. There are many definitions of inflammation with various levels of complexity. We define inflammation as an innate immune system-mediated process that is governed by the proinflammatory cytokines and chemokines, such as TNF-α, IL-1α, IL-1β, IL-6, IL-18, GM-SCF, IL-8, and MIP1a. As a result, the robust inflammatory response is associated with the increased expression of proteins in enzymatic pathways, which leads to the release of cytotoxic molecules, including nitric oxide (NO), reactive oxygen species (ROS), prostaglandins, and an array of proteases. The fundamental role of inflammatory responses is to eliminate invading pathogens and to help an organism recover from tissue damage. Therefore, the immunological responses to infection or tissue injury are often associated with the release of potent antimicrobial components. Although inflammatory responses are crucial for host-survival (1), high concentrations of cytotoxic molecules lead to damage in surrounding tissues, which perpetrates further injury (1).

In an exposed organism, the initial innate immune response defines the outcome of the adaptive immune response. The adaptive immune response is designed to fine-tune and increase the efficacy of inflammation in clearing pathogens, speeding up resolution of infection or injury, and promoting wound healing. The adaptive and innate immune responses are guided by the expression profile of proteins that sense the environment and provide the necessary information to various immune cell subsets to orchestrate fast and efficient return to homeostasis. These proteins recognize specific molecular patterns and, thus, were named pattern recognition receptors (PRRs) (2, 3).

Molecular patterns that are recognized by the PRRs are broadly categorized into two groups: (1) those that accompany pathogens/microbes are called pathogen-associated molecular patterns (PAMPs) or microbial-associated molecular patterns and (2) those that are released by the injured tissues of dying cells called danger-associated molecular patterns (DAMPs). As of now, several families of PRRs were identified, including toll-like receptors (TLRs), C-type lectin receptors (CLRs), RIG-I-like receptors (RLRs), and nucleotide-binding oligomerization domain (NOD)-like receptors (NLRs) (2). These PRRs play important roles in regulation of tissue inflammation.

Toll-like receptors are transmembrane proteins that are expressed in most cell types, either at the cell surface (TLR1, 2, 4, 5, 6, 10) or in endosomes (TLR3, 7, 8, 9). They can detect a variety of molecules, including proteins, lipopeptides, and nucleic acids (single-stranded RNA, double-stranded RNA, or CpG DNA). Ligand detection by TLRs initiates intracellular signaling cascades that activate inflammatory mediators, such as interferon regulatory factor (IRF) family members or NF-κB (4). CLRs are another family of PRRs that bind to carbohydrate structures, including mannose, fucose, and glucan on pathogens. They are mainly expressed on the surface of antigen-presenting cells (APCs), such as monocytes, macrophages, and dendritic cells (DCs) (5). The binding of pathogens to CLRs leads to its internalization, degradation, NF-κB activation, and subsequent antigen presentation to the T cells. Alternatively, RLRs are cytosolic PRRs that are expressed by both immune and non-immune cells that sense cytoplasmic RNA. During a viral infection, RLRs recognize viral dsRNA in the cytoplasm and activate antiviral signaling pathways, including Type I interferon and NF-κB. There are three members in RLRs family: RIG-I, melanoma differentiation-associated gene 5 (MDA5), and laboratory of genetics and physiology 2 (6).

Nucleotide-binding oligomerization domain-like receptors are the most recently discovered group of PRRs (7, 8). They were first described in plants, where they were shown to provide protective immunity against infection. As a protection mechanism, plants employ PRRs, such as intracellular immune receptors termed nucleotide-binding site leucine-rich repeat (LRR) proteins, which are structurally similar to mammalian NLRs. The importance of NLRs in regulating inflammation is highlighted by their evolutionary conservation across vertebrate species and the association of genetic mutations in several NLR genes with autoinflammatory diseases (9). NLRs were previously grouped under the term CATERPILLAR [Caspase-recruitment domain (CARD) transcription enhancer, R (purine)-binding, pyrin, lots of leucine repeats] gene family (10). Other research groups have named these proteins NOD-LRR family and NACHT [domain present in NAIP, class II transactivator (CIITA), HET-E, and TP1]-LRR family (8, 10). The study of NLR gene family emerged in the early 2000s following the discovery of their structural similarity to the CIITA, which is the master regulator of MHC class II transcription (11). NLR genes quickly surfaced as important mediators in apoptosis, immune responses, and inflammatory diseases. Currently, NLRs include 23 members in humans and at least 34 members in mice (12).

**Figure 1:**
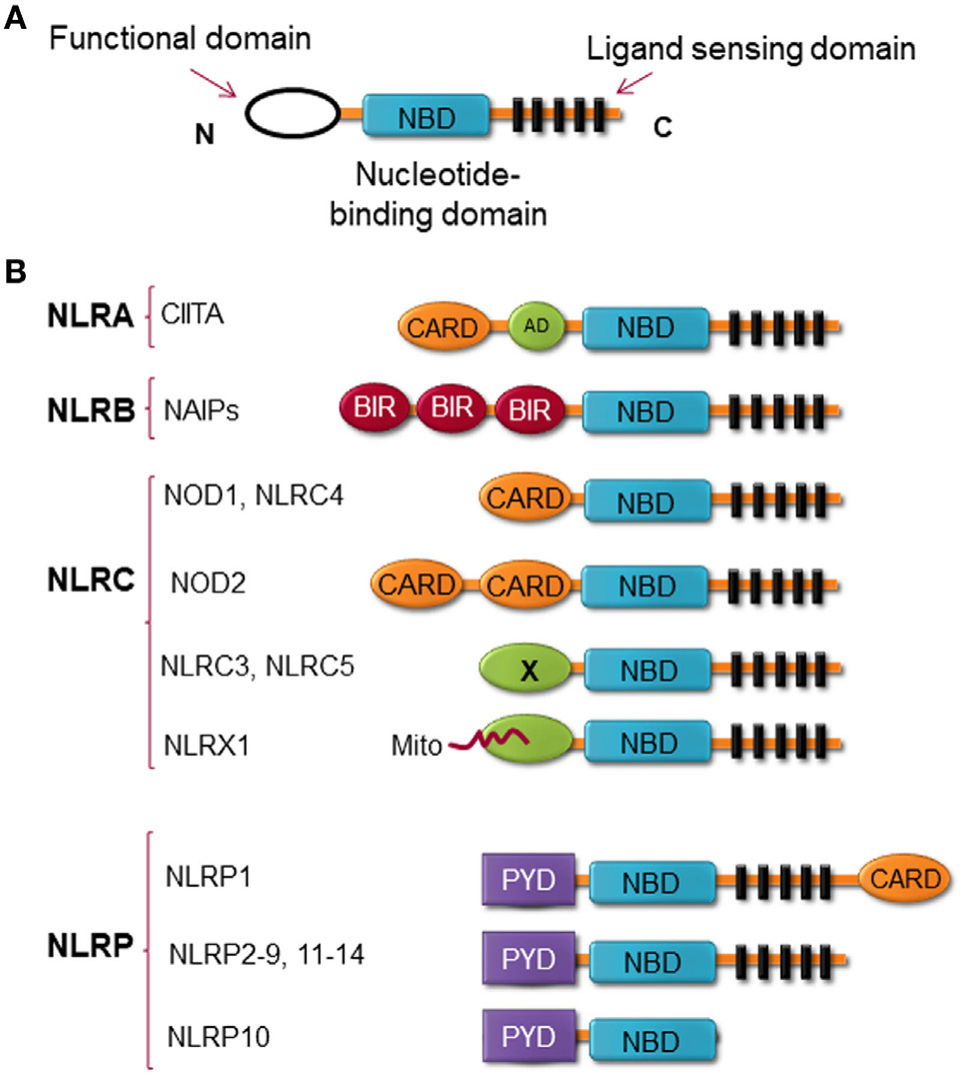
Nucleotide-binding oligomerization domain (NOD)-like receptors (NLRs) structure. **(A)** The general structure of NLRs, consist of three domains, including functional domain, nucleotide binding and oligomerization domain, and ligand sensing domain. **(B)** Classification of NLRs based on the nature of their functional domain: NLRA, an acidic transactivation (AD) domain; NLRB, a baculovirus inhibitor of apoptosis protein (IAP) repeat (BIR); NLRC, a caspase-recruitment and activation domain (CARD); and NLRP, a pyrin domain (PYD). In NLRC subfamily, the X displays an unknown domain that has no homology with the other NLR members. Mito is the mitochondria-localization sequence that directs NLRX1 to the mitochondria.

Structurally, NLRs consist of three highly conserved domains with the C-terminal region leucine-rich repeat (LRR), which is thought to be responsible for ligand binding; the central nucleotide binding ATPase domain NACHT/NBD (also known as NOD), which promotes oligomerization and activation; and the N-terminal domain, which contains either a CARD or pyrin domain (PYD) and is responsible for protein–protein interaction (13) (Figure 1). NLRX1 is an exception to this rule, instead of expressing an N-terminal protein-protein interaction domain, it possesses mitochondria-localization sequence (14). NLRs can be categorized by their structure and by their function. By the structure of the N-terminal domain, members of the NLR family are categorized into at least four subfamilies, including (1) NLRAs are characterized by the expression of acidic transactivation domain, (2) NLRBs contain baculovirus inhibitor of apoptosis protein repeat (BIR), (3) NLRCs possess CARD or an undefined domain, and (4) NLRPs contain PYD (Figure 1) (15–17).

**Figure 2:**
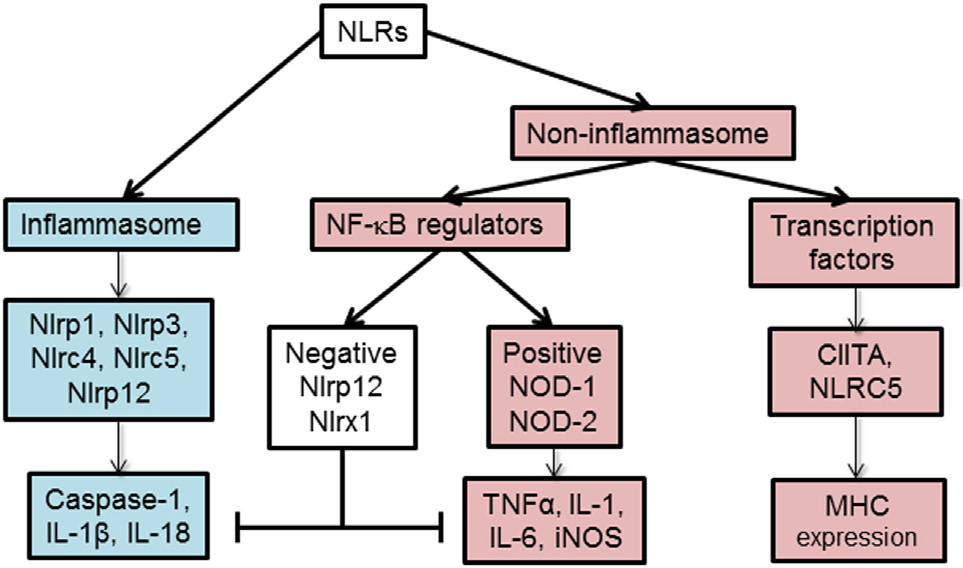
Functional characterization of nucleotide-binding oligomerization domain (NOD)-like receptors (NLRs). NLRs can be classified depending on their mechanism of action to inflammasome and non-inflammasome forming NLRs. The inflammasome forming NLRs assemble inflammasome that activates caspase-1 and promotes the production of inflammatory cytokines, IL-1β and IL-18. In the group of non-inflammasome forming NLRs, some NLRs regulate MHC II expression, while other NLRs regulate NF-κB signaling. The regulators of NF-κB consist of NLRs that enhance (NOD-1, NOD-2) or inhibit (NLRP12, NLRX1) NF-κB signaling pathway. The negative NLRs, NLRP12 and NLRX1, can inhibit both inflammasome-dependent and -independent cytokine production. NLRC5 and NLRP12 have been described to influence both inflammasome and non-inflammasome signaling pathways in a cell- and stimuli-dependent fashion.

Based on their function, NLRs can be classified into two main categories, non-inflammasome and inflammasome forming as depicted in Figure 2. Non-inflammasome NLRs can be further categorized into NF-κB regulators and transcription factors. Some NLRs, such as NLRP12, have been reported to play anti-inflammatory and proinflammatory roles depending on the experimental condition or the type of stimuli. Additionally, some NLRs act as transcription factors, such as CIITA and NLRC5, that indirectly regulate the immune response by tuning the expression of MHC II and I on APCs (18).

In this review, we provide an overview of the role of NLRs in inflammation during MS. The main focus of the review is on the innate immune response with the special emphasis on negative regulators of inflammation. Although the majority of research is devoted to the stimulators of inflammation, in our opinion, the endogenous inhibitors of inflammation have a promising future as therapeutic targets for inflammatory disorders.

## Multiple Sclerosis

Multiple sclerosis is a devastating pathology that is diagnosed in young people predominantly between the ages of 20–40. Among neurological diseases, MS is the most common cause of disability in young adults (19). It is accompanied by the progressive decline of neurological functions, including vision and cognitive impairments and deterioration of sensory and motor functions (20).

Multiple sclerosis is a lymphocyte-mediated autoimmune disease (19). Indeed, ablation of adaptive immunity or inhibition of lymphocyte migration has been shown to slow down or even reverse the course of MS. However, such extreme therapies are associated with increased risk of fatal infections due to immunosuppression (21). Interestingly, all people possess autoreactive lymphocytes that patrol the central nervous system (CNS) during bacterial and viral infections, but only a relatively small number of them develop MS. This suggests the existence of predisposing factors other than lymphocytes-associated factors in MS patients.

Epidemiological studies suggest that MS results from the contribution of both genetic and environmental factors (22). Interracial studies and studies with monozygotic twins strongly suggest a genetic component of the disease, while geographical and migratory studies point to the involvement of environment in the MS pathology (23). The point of consensus between environmental and genetic theories of the etiology of MS is that in all cases immune system is deregulated. However, what remains uncertain is which components of the immune system and inflammatory response are the contributors and which are the result of the disease process. A distinguishing feature of MS pathology from other inflammatory diseases are the MS plaques, also known as lesions, which are widely spread throughout the CNS, particularly in the periventricular white matter, optic nerve, brain stem, and spinal cord areas. Pathological features of these plaques, include oligodendrocyte cell death, myelin destruction, axonal damage, glial scar formation, disruption and leakage of the blood–brain barrier (BBB), and the presence of inflammatory infiltrates composed of autoreactive T lymphocytes, microglia, macrophages, astrocytes, B lymphocytes, and ependymal cells (24–26). Activated macrophages, microglia, and astrocytes have been described in demyelinating lesions and are believed to play key roles in perpetuating disease progression in later stages of the disease (27–29). Most of the pathophysiological features of MS are reproduced in an rodent model called experimental autoimmune encephalomyelitis (EAE). It is based on immunization of animals with CNS antigens, including immunodominant peptides such as MOG35-55 and PLP (30). Although most of the pathophysiological changes were well characterized, behavioral description of EAE remains underdeveloped and includes clinical scores that quantify the degree of ascending paralysis (31). Despite being widely criticized as being different from human MS, EAE model helped in developing multiple disease modifying drugs (30).

### Innate Immune Response

Several studies from different laboratories suggest that CNS immune cells are activated before the appearance of clinical symptoms of MS and before T cell infiltration. For example, work from Dr. Fabry’s group demonstrated activation of microglia and CNS DCs ahead of the infiltration of MOG-specific T cells in the olfactory bulb (32, 33), cerebellum, and along the white matter tracts (34). The activation of these cells was specific to EAE and was significantly increased compared to healthy mice and CFA-injected controls. Interestingly, an increase in microglia and DCs facilitates the migration of lymphocytes within the brain, which suggests that activation of these cells potentiate the effect of pathogenic T cells. Dr. Pham’s (35) group and others demonstrated that inflammatory cells may utilize the rostral migratory pathway that is used by stem cells that go to the olfactory bulb. These observations put emphasis on early inflammatory events that precede the T cell infiltration and appearance of symptoms, which indicates that the activation of innate immune response potentiates CNS inflammation and may play a role in development of aberrant T cell responses.

Experimental autoimmune encephalomyelitis studies demonstrate that astrocytic responses coincide with early axonal damage (36). Astrocyte mediated inflammation is associated with inflammatory responses that are characterized by robust proliferation and hypertrophy of astrocytes that is termed astrogliosis. Astrocytes maintain the integrity of the BBB, provide for the energy needs of neurons, and are responsible for rapid reuptake of glutamate (37). Similar to macrophages/microglia, astrocytes express molecular machinery, which enables them to regulate inflammation and adaptive immune responses within the CNS (38–40). Therefore, astrocytes have all the features to orchestrate both an inflammatory response within the CNS and to regulate the influx and activity of lymphocytes. Astrocytes have been shown to upregulate inducible nitric oxide synthase (iNOS) in response to the elevated levels of proinflammatory cytokines (41). Increased iNOS activity generates NO, which is associated with the production of cytotoxic nitrites and nitrates that impede astrocyte-dependent glutamate uptake resulting in CNS damage (41). Activated astrocytes release inflammatory cytokines, such as IL-6, IL-1β, and TNF-α, that affect the tight junctions of endothelial cells, which allows the passage of immune cells through the BBB. Moreover, activated astrocytes secrete chemokines, such as MCP-1, RANTES, IP-10, SDF-1, and IL-8, which recruit leukocytes, such as monocytes, neutrophils, DCs, and lymphocytes, from the periphery to the CNS parenchyma, which further contribute to the cytotoxicity of the micro-environment (42). Proinflammatory cytokines and chemokines also activate microglia, which are the CNS-resident immune cells (43).

Microglia constantly monitor CNS environment and orchestrate innate immune response within the CNS parenchyma (44). Microglia originate from embryonic yolk sac at a very early stage of development, seed the brain, and stay there into adulthood (45–47). The morphology of microglia differs from that of conventional macrophages due to the presence of highly motile projections. Activated microglia have increased ability of phagocytosis and antigen presentation within the CNS (48–50). For a long time, activated microglia were considered to be indistinguishable from activated macrophages. Recently this notion was challenged; and TMEM119 emerged as a microglial marker (51). In many CNS pathologies, including MS, the number of microglia often increases in a phenomenon that is called reactive microgliosis (52, 53). Microglia release inflammatory mediators, such as iNOS, TNF-α, IL-1β, IL-6, IL-12, which aid in the recruitment of adaptive immune cells into the CNS (54).

Dendritic cells are professional APCs that uptake the antigen and travel to the local lymph node. Unlike other organs in the body, such as liver, skin, or intestine, the CNS parenchyma has a low number of DCs in the steady state (55). However, a recent work using the developmental and functional criteria demonstrated that DCs develop from their precursors (pre-DCs) in the meninges and choroid plexus of mice (56). In the case of neuroinflammation due to injury or infection, the BBB gets compromised and peripheral DCs infiltrate the CNS (55), where they contribute to antigen presentation and reactivation of encephalitogenic T cells (34, 57). Several studies also demonstrated the accumulation of DCs in white matter lesions and cerebrospinal fluid (CSF) of MS patients (58, 59).

### Adaptive Immune Response

The role of T lymphocytes in MS pathogenesis has been well established (60, 61). After crossing the BBB, activated autoreactive T cells secrete inflammatory cytokines that activate macrophages and microglial cells. In turn, macrophages and microglia secrete chemokines that contribute to the recruitment of other T cells, DCs, and macrophages, which further amplifies the ensuing inflammatory cascade within the CNS. Furthermore, recruited T cells are activated by local APCs (62). Numerous CD4^+^ T cell subsets have been implicated in MS, including T helper 1 (T_H_1) and T helper 17 (T_H_17) being the key components in the inflammatory response (63). T_H_1 differentiation is favored in the presence of IL-12. Once T_H_1 cells are activated, they release proinflammatory IFN-γ, TNF-α, GM-CSF, and IL-2 cytokines (62). T_H_17 differentiation and development occur in the presence of IL-1, IL-23, IL-6, and TGF-β. Activation of this subtype of CD4^+^ T helper cells results in secretion of IL-17A, IL-17F, IL-9, IL-21, IL-22, TNF-α, and GM-CSF (62, 64). Furthermore, CD8^+^ T cells are also implicated in MS and are primarily found in the outer boundary of the lesions and in the perivascular area (62, 64). Interestingly, CD4^+^ T cells were shown to play a role in the initial stages of lesion formation, whereas CD8^+^ T cells were shown to be involved in the amplification of the inflammatory response, which resulted in damages (62, 63, 65). During inflammation, B cells and plasma cells are also recruited to the CNS. Plasma cells produce specific antibodies to myelin antigens that initiate the complement cascade, leading to destruction, opsonization, and subsequent phagocytosis of the myelin sheath (66).

### Inflammatory Nature of MS

**Figure 3:**
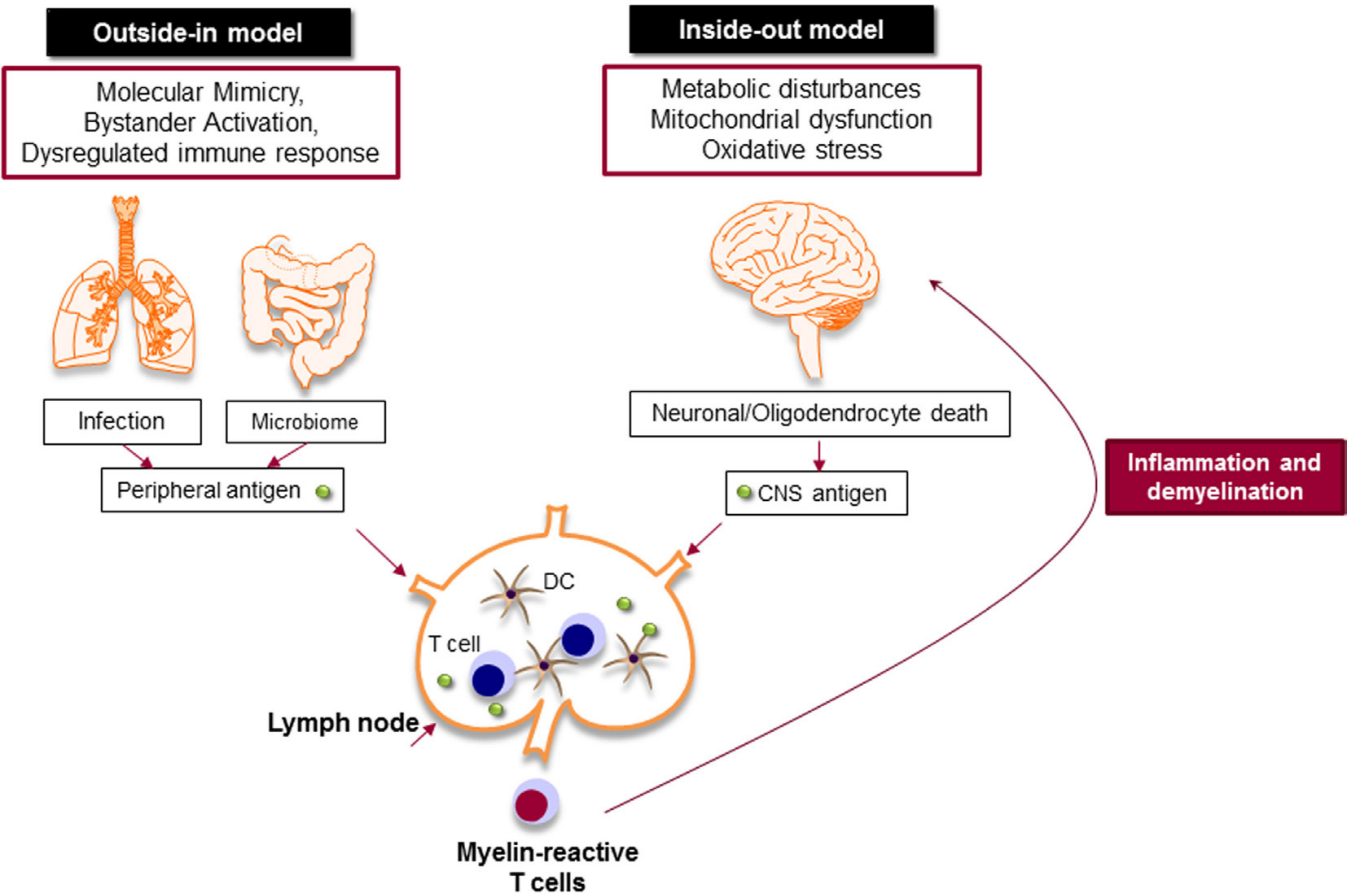
Myelin-specific T cells are activated in the periphery by peripheral antigens or the central nervous system (CNS) antigens. In outside-in model, cross-activation of T cells by pathogen-derived molecules (molecular mimicry) or non-specific activation of T cells by superantigens (bystander activation) might be involved in the activation of myelin-specific T cells. The gut microbiome consists of digestive-tract associated microbes is also important to balance and regulate the immune response. The activated T cells attack CNS and cause inflammation and neurodegeneration. Inside-out model argues that the CNS inflammation primarily begins in the absence of a direct immune attack, in which neuronal/oligodendrocyte injury releases CNS antigen that triggers the immune response in the periphery.

Despite extensive efforts to define MS immunopathology, the origin of the disease is still a matter of debate. The presence of autoreactive T and B cells in the CNS strongly supports the hypothesis that MS is primarily caused by an aberrant immune response against the CNS antigens, particularly myelin, in which chronic immune responses cause oligodendrocyte death and progressive demyelination (outside-in model of MS) (Figure 3) (67). It is still unknown how myelin-specific T cells are activated in the periphery. There are studies that support the activation of myelin-specific T cells by infectious agents (molecular mimicry) or non-specific T cells by superantigens (bystander activation) (68). Pathogens, such as *Mycoplasma pneumoniae* and *Chlamydia pneumoniae*, viruses, such as *Epstein Barr virus* and human *herpes virus*, and enterotoxins produced by *Staphylococcus aureus* are shown to be associated with the development or exacerbation of MS (69). The gut microbiome, which consists of digestive tract-associated microbes, actively regulate the homeostasis of the immune system. It has been suggested that dysbiosis may lead to dysregulation of the immune responses both in the periphery and the CNS (70).

Inside-out model of MS presents the idea that MS is primarily initiated by a neurodegenerative event (Figure 3). In this model, the oligodendrocyte injury or death would be the trigger of the CNS inflammation that presumably begins in the absence of a direct immune attack. Oligodendrocytes are extremely vulnerable to the oxidative stress due to their high metabolic rate, large intracellular iron stores, and low levels of antioxidative enzymes. Exposure to stress reactions or metabolic disturbances can lead to caspase activation and subsequent oligodendrocyte death (71). Oxidative stress also results in mitochondrial dysfunction, which causes axonal damage and oligodendrocyte apoptosis. As a result, myelin antigens are released into the peripheral circulation and activate autoreactive T and B cells that migrate to the CNS and induce inflammatory cascade.

**Figure 4:**
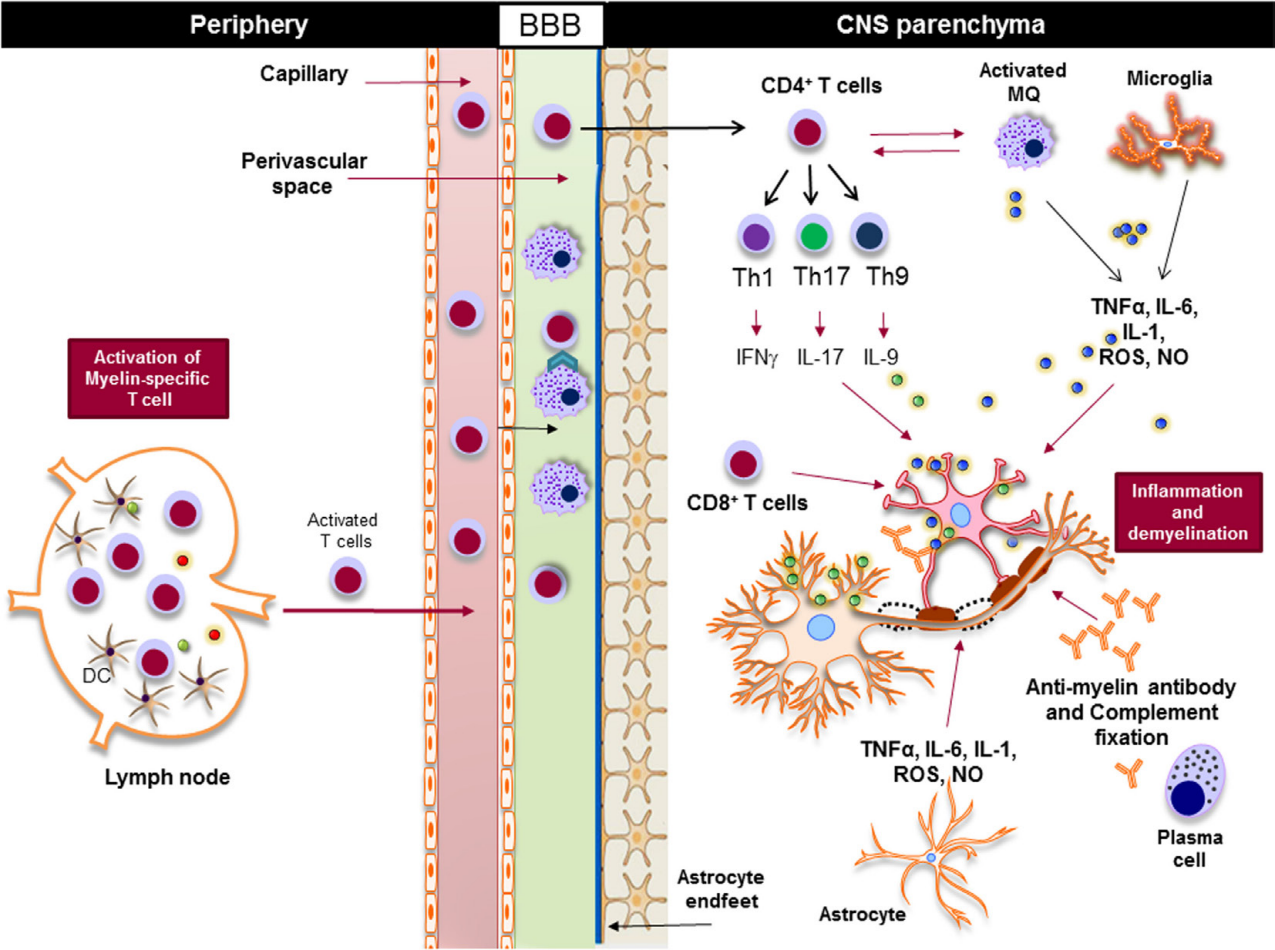
Innate and adaptive immunity in the pathogenesis of multiple sclerosis. Myelin-reactive T cells are activated in the periphery and accumulate in the perivascular spaces, where they are reactivated by the central nervous system (CNS) myeloid cells, such as macrophages, and enter the CNS parenchyma. CD4^+^ T cells are differentiated to different inflammatory subsets, such as Th1, Th17, and Th9, and once in the CNS they promote the activation of CNS-resident innate immune cells, such as macrophages and microglia. Inflammatory mediators, such as TNF-α, IL-6, IL-1, reactive oxygen species (ROS), and nitric oxide (NO), released from activated macrophages, microglia, and astrocytes damage oligodendrocytes and neurons, leading to demyelination. Activated cytotoxic CD8^+^ T cells directly induce apoptosis in oligodendrocytes *via* FAS/FASL interaction, while plasma cells produce antimyelin antibodies that activate the complement system and damage oligodendrocytes.

Regardless of the nature of the primary trigger, both innate and adaptive immune responses are involved in potentiating demyelinating neuroinflammatory disease in MS (Figure 4). Although the infiltration of lymphocytes into the CNS is more prominent in the early stages of the disease, the disease becomes less dependent on lymphocytes and more neurodegenerative in later stages. Inflammation is present at all stages of the disease; it is triggered either by the infiltration of peripheral immune cells into the CNS or by the CNS-resident cells that respond to the CNS insult. From a classical point of view, NLRs are responsible for rapid sensing of PAMPs, such as products of microbial metabolism, and DAMPs, such as uric acid, ATP, nucleic acid, and ROS (72–75). The roles and functions of NLRs span beyond sensing of PAMPs and DAMPs; they are highly involved in the regulation of inflammatory pathways, such as NF-κB and mitogen-activated protein kinases (MAPK) (75–77). Next, we discuss positive and negative effect of NLRs on CNS inflammation (Table 1).

**Table 1:** The role of inflammatory and anti-inflammatory nucleotide-binding oligomerization domain (NOD)-like receptors (NLRs) in multiple sclerosis (MS) patients and mouse MS models.

		Subjects	Major findings	Ref.
Inflammatory NLRs	NLRP1	MS patients	A homozygous missense variant in NLRP1 (Gly587Ser) was associated with familial MS	(94)
		MS patients	Compound heterozygous mutation was observed in several MS patients	(95)
	NLRP3	EAE	*Nlrp3^−/−^* mice developed ameliorated EAE, associated with a significant reduction of the inflammatory infiltrate to the CNS and lower production of IL-18, IFN-γ, and IL-17	(99)
		EAE	*Nlrp3*^−/−^ mice were resistant to EAE with decreased inflammatory cell infiltration to the CNS. The activation of NLRP3 inflammasome in APCs is crucial for T cell migration to the CNS	(105)
		EAE	High-dose adjuvant induced severe EAE and neuronal damage in *Nlrp3^−/−^* mice, which was inflammasome-independent and resistant to IFN-β therapy	(108)
		MS patients	IFN-β treatment attenuated the course and severity of MS by reducing the activity of NLRP3 inflammasomes	(112)
		MS patients	Q705K polymorphism (rs35829419) results in overactive NLRP3 inflammasome, which was associated with IFN-β response in MS patients	(112)
	NLRC4	Cuprizone mouse model	NLRC4 inflammasome in microglia and astrocytes is associated with neuroinflammation and demyelination	(115)
	NOD1 and NOD2	EAE	*Nod1*^−/−^ and *Nod2*^−/−^ mice were highly resistant to EAE. Reduced number of activated APC and activation of T cells in the CNS were observed	(120)
Anti-inflammatory NLRs	NLRP12	EAE	*Nlrp12^−/−^* mice developed EAE earlier with more severe clinical and pathological outcomes. The absence of Nlrp12 results in an increased inflammatory response in microglia	(126)
		EAE	*Nlrp12^−/−^* mice had ameliorated EAE course with atypical symptoms, including ataxia and impaired balance control, which was associated with increased production of IL-4	(128)
	NLRX1	EAE	Protective role of NLRX1 in EAE. *Nlrx1^−/−^* mice showed increased macrophage/microglial activation and cytokine production, which resulted in increased tissue damage	(155)

## NLRs as Positive Regulators of Inflammation

**Figure 5:**
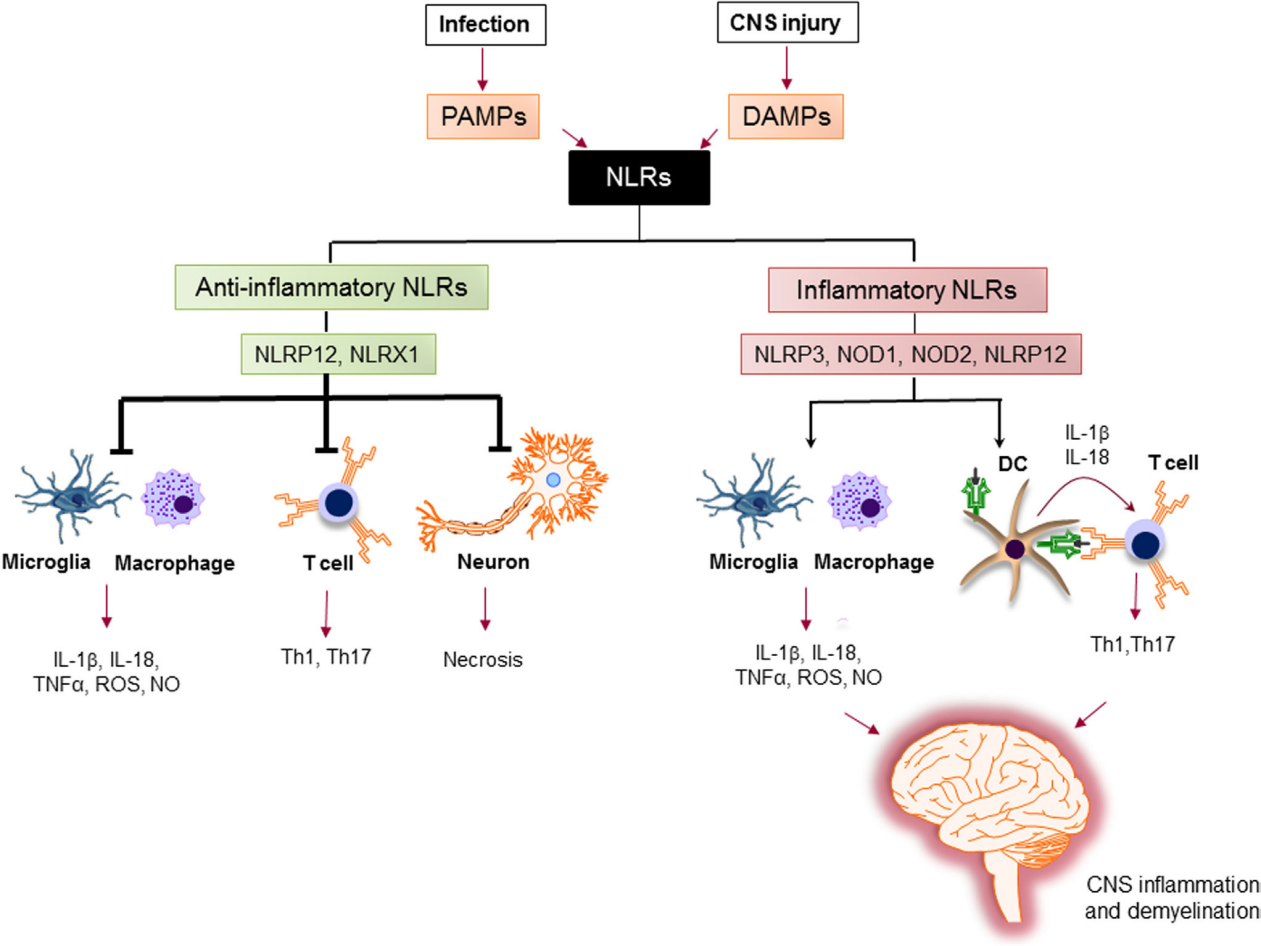
The role of nucleotide-binding oligomerization domain (NOD)-like receptors (NLRs) in regulation of inflammation in multiple sclerosis (MS). Pathogen-associated molecular patterns (PAMPs) and danger-associated molecular patterns (DAMPs) trigger the immune response *via* NLRs. Upon sensing ligands, inflammatory NLRs not only initiate inflammatory response in innate immune cells, such as macrophages and microglia, but also bridge the immune response from innate to adaptive immune response *via* instructing T cell response by dendritic cells (DCs) to generate different subsets of pathogenic T helper subsets (e.g., Th1, Th17). On the other hand, anti-inflammatory NLRs inhibit the production of inflammatory mediators by macrophages and microglia, suppress the differentiation of T cells to inflammatory subsets, and protect neurons from necrosis. Thereby, the increased activation of inflammatory NLRs and the impaired function of anti-inflammatory NLRs lead to central nervous system inflammation and demyelination in MS.

The activation of proinflammatory NLRs, such as NLRP1, NLRP3, NLRP6, NLRP7, NLRP12, NLRC4, and NAIP has been reported to result in the formation of inflammasome and production of potent inflammatory cytokines, such as IL-1β and IL-18 (78, 79). The assembly of inflammasome consists of binding of a regulatory NLR to ASC-adaptor molecule and an inactivated form of caspase-1. The formation of inflammasome activates caspase-1, which cleaves pro-IL-1β and pro-IL-18. The cleavage of IL-1β and IL-18 is necessary for their secretion (80). Activation of IL-1β is an essential innate immune cytokine, which is released primarily by myeloid cells in the CNS. It is involved in the leukocyte infiltration primarily by inducing the expression of cytokines, chemokines, and adhesion molecules (81). IL-18 is produced by a variety of cells, including monocytes, macrophages, microglia, and astrocytes. It plays a role in the recruitment of polymorphonuclear leukocytes by upregulating the expression of intracellular molecule-1 on endothelial cells (82). Activated caspase-1 has been shown to be significantly increased in MS patients and in EAE (83, 84). The role of inflammatory NLRs in the immunopathogenesis of MS is summarized in Figure 5.

### NLRP1

NLRP1 was the first discovered inflammasome (85). It consists of NLRP1, ASC, the cysteine proteases caspase-1 (86). NLRP1 differs from other NLR proteins in that it has two signal transduction domains: a PYD and CARD (Figure 1) (87). NLRP1 is highly expressed by immune cells and is present at low levels in all tissues (88). In the CNS, expression of NLRP1 is highly dynamic and changes rapidly during various pathologies, such as trauma and stroke (86, 89). NLRP1 is expressed by neurons, microglia, and astrocytes and was shown to play a major role in neuronal death and CNS inflammation (90). There is strong evidence that suggests a link between NLPR1 and autoimmunity. Several studies found an association between NLRP1 and vitiligo (91), autoimmune thyroid diseases (92), and type I diabetes (93). In a recent publication, Maver et al. linked homozygous missense variant in NLRP1 gene (Gly587Ser) with familial forms of MS (94). Also, Bernales et al. found several *NLRP1* compound heterozygote mutations in MS patients (95). The association of NLRP1 and the pathophysiology of MS needs further investigation.

### NLRP3

Given its prominent role in a number of diverse diseases, NLRP3 is by far the most well-known activator of inflammasome signaling. Mutations in NLRP3 gene lead to several autoinflammatory disorders referred to as the cryopyrin-associated periodic syndromes (96). NLRP3 is activated in two steps. The first step is priming the cells by PAMPs or DAMPs *via* TLRs, which leads to the activation of NF-κB signaling that triggers the expression of inflammasome-related components, including NLRP3, pro-IL-1β, and pro-IL-18. The second step is oligomerization of NLRP3 and its association with an adaptor protein ASC and pro-caspase-1. This complex triggers the activation of caspase-1 that cleaves pro-IL-1β and pro-IL-18 into their mature and secreted forms: IL-1β and IL-18 (97). Activation of the NLRP3 inflammasome also results in pyroptosis, a caspase 1-dependent cell death, which is a highly inflammatory. Pyroptosis results in cell lysis and the release of cytosolic components into the extracellular environment (98).

Previous studies demonstrated that NLRs and their adaptors could positively influence the development and the severity of EAE (99). Deletion of *Nlrp3, ASC*, or the *caspase-1* gene resulted in protection against EAE (99, 100). NLRP3 causes severe inflammatory symptoms in EAE by producing more IL-1β and IL-18, which stimulate the development and activation of T_H_1/T_H_17 cells and enhance their infiltration into the CNS (99). It was shown that NLRP3 inflammasome assembles in human CD4^+^ T cells and initiates caspase-1 activation and IL-1β production, which results in promoting IFN-γ secretion and T_H_1 differentiation in an autocrine manner (101). Other studies demonstrate key roles for the inflammasome-mediated IL-1 production in the induction of GM-CSF by both T_H_1 and T_H_17 cells in EAE (102). In an alternative pathway, NLRP3 inflammasome engages caspase-8 instead of caspase-1 (103). The importance of NLRP3-caspase-8 inflammasome was recently shown in the production of IL-1β by T cells that support the survival of T_H_17 cells in EAE (104). Moreover, NLRP3 inflammasome in APCs played a critical role in upregulating chemotactic proteins, such as osteopontin, CCR2, and CXC chemokine receptor (CXCR) 6, in T_H_1 and T_H_17 cells, thereby inducing T cell migration to the CNS in EAE (105).

Demyelinating neuroinflammatory disease was shown to develop in the absence of NLRP3 inflammasome, which resulted in more severe EAE (100, 106). These studies showed that the induction of potent innate immune responses with high dosages of heat-killed mycobacteria (Mtb) in adjuvants drives aggressive neuronal damage and EAE disease in mice that are deficient in either ASC or NLRP3 (107). Disease progression in this more aggressive model of EAE (referred to as type B EAE) was found to be dependent on membrane-bound lymphotoxin-β receptor (LTβR) and CXCR2 (108).

Many reports demonstrate detrimental role of NLRP3 inflammasome in MS patients. The expression of caspase-1 and IL-18 are elevated in peripheral mononuclear cells from MS patients compared to those from healthy controls (83). Moreover, the levels of IL-1β are upregulated in CSF of MS patients and correlate with the progression of MS (109). MS treatments, such as glatiramer acetate and IFN-β, elevate the levels of endogenous IL-1 receptor antagonist in MS patients (110, 111). A study by Malholtra et al. showed that the IFN-β treatment attenuated the course and severity of MS by reducing the activity of NLRP3 inflammasomes *via* suppressing caspase-1 dependent IL-1β secretion (112). The Q705K polymorphism (rs35829419) in exon 3 produced an overactive NLRP3 inflammasome, which was associated with IFN-β response in MS patients (112).

Beyond its role in forming inflammasome, NLRP3 is a transcriptional regulator of T_H_2 differentiation in a T cell-intrinsic manner. A recent study by Bruchard et al. showed that NLRP3 acts as a key transcription factor in T_H_2 differentiation in conjunction with IRF4. NLRP3 binds and activates *IL-4* promoter in T_H_2 cells in an inflammasome-independent manner (113). The T cell-intrinsic role of NLRP3 needs further investigation.

### NLRC4

NLRC4 is well characterized in bacterial infection, such as *Salmonella typhimurium* and *Legionella pneumophila* (114). In sterile inflammation, the CNS-associated DAMP, lysophosphatidylcholine, activates NLRC4 inflammasome in microglia and astrocytes. A recently published study revealed that the activation of NLRC4 inflammasome in microglia and astrocytes is associated with neuroinflammation and demyelination in cuprizone mouse model (115), a model of toxin-induced demyelination without the activated adaptive immunity (116). The increased NLRC4 expression in the lesions of human MS brains confirms the association between NLRC4 and neuroinflammation in MS (115).

### Other Proinflammatory NLRs

Other proinflammatory NLRs, such as NOD1, NOD2 (117), and NLRP10 (118), induce inflammation, which is independent of inflammasome formation. These NLRs upregulate NF-κB and activate MAPK pathways (119). Furthermore, NOD2 interacts with mitochondrial antiviral signaling protein (MAVS) (77), which is essential for the production of IFN-β to suppress virus replication during viral infections. Shaw et al. showed that *Nod1*^−/−^ and *Nod2*^−/−^ mice are highly resistant to EAE due to the reduced number of activated APC, which leads to a reduced activation and expansion of T cells in the CNS (120). These findings collectively demonstrate that NLR proteins can exacerbate MS, either *via* formation of inflammasome or stimulation of inflammatory pathways, such as NF-κB and MAPK.

## NLRs as Negative Regulators of Inflammation

The activation of PRR by PAMPs and DAMPs is negatively regulated by members of NLRs family, including NLRP12, NLRX1, and NLRC3. The role of anti-inflammatory NLRs in regulation of inflammation in MS is summarized in Figure 5.

### NLRP12

NLRP12 is a pyrin-containing NLR protein that is expressed in cells of myeloid origin and is formerly known as RNO, PYPAF7, and Monarch-1 (121, 122). The HUGO Gene Nomenclature Committee approved the name of NLRP12 for this gene. Two research groups simultaneously cloned the full-length sequence of human NLRP12 (121, 122) and, later, it was identified in HL60 human leukemic cell line (123). In humans, NLRP12 is expressed in neutrophils, eosinophils, macrophages, monocytes, and immature DCs (123–125).

Since the discovery of NLRP12, there have been contrasting reports that demonstrated both proinflammatory and anti-inflammatory roles of NLRP12 in cell-type and stimuli-specific manners (126–130). Early studies showed that NLRP12 is an inflammatory NLR that interacts with ASC to form inflammasome, leading to caspase-1 activation and release of mature IL-1β. Evidence for the involvement of NLRP12 in inflammasome formation and activation are largely derived from *in vitro* studies that used overexpression systems (121). Recent studies showed the role of NLRP12 in activation of inflammasome by intracellular pathogens, such as *Yersinia Pestis* and *Plasmodium* infection (130, 131), but not by other pathogens, such as *Salmonella, Klebsiella, Escherichia, Mycobacterium*, and *Listeria* species (127, 132, 133). Taken together, these studies established a biologically relevant role for the NLRP12 inflammasome in innate immune responses against pathogens; however, the exact molecule that triggers NLRP12 inflammasome remains unknown.

Alternatively, there are studies that identified NLRP12 as a negative regulator of inflammation that inhibits NF-κB signaling in innate immune cells. It was shown that the activation of human peripheral blood granulocytes and monocytes by TLR4 or TLR2 agonists (*E. coli* LPS or synthetic lipopeptide Pam3Cys, respectively) reduces the expression of NLRP12 (122). Moreover, the expression of NLRP12 declines significantly in myeloid cells (THP-1 human monocytic cell line) after *in vitro* stimulation with live bacteria, such as *Mycobacterium* or *Plasmodium*, or cytokines, such as TNF-α or IFN-γ (134). When *NLRP12* was knocked down in THP1 cells using shRNA, the expression levels of proinflammatory cytokines significantly increased following LPS or *M. tuberculosis* treatment (134). A transcriptional repressor called B lymphocyte-induced maturation protein-1 is induced by TLR stimulation and downregulates NLRP12 expression by binding to *Nlrp12* promoter and recruiting histone deacetylases (135, 136).

**Figure 6:**
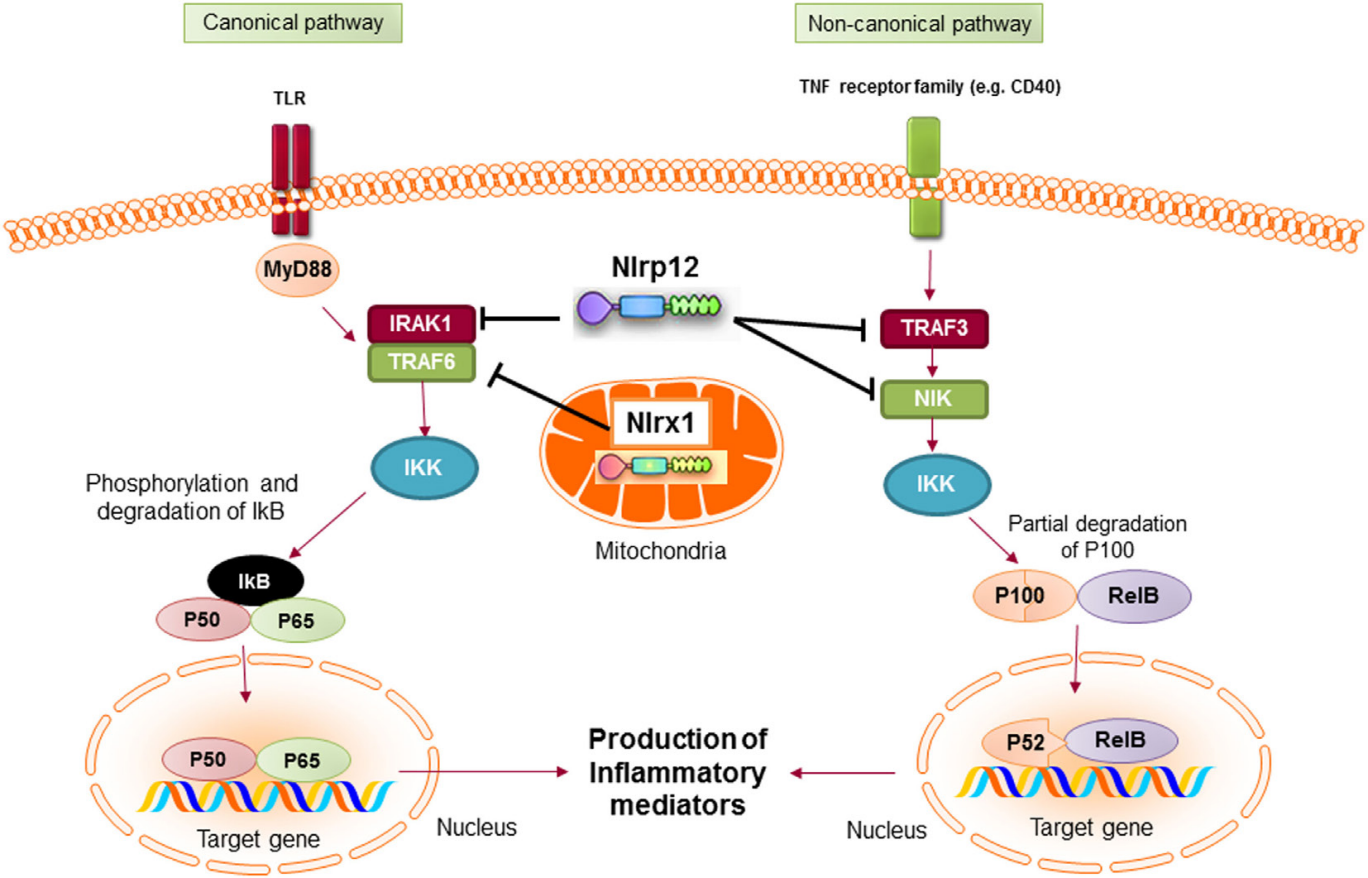
Inhibitory nucleotide-binding oligomerization domain (NOD)-like receptors put the brake on NF-κB activation *via* canonical and non-canonical pathways. Both NLRX1 and NLRP12 inhibit the activation of NF-κB canonical pathway following toll-like receptor (TLR) stimulation. Nlrx1 interacts and inhibits TNF receptor-associated factor (TRAF) 6, while NLRP12 inhibits the phosphorylation of IRAK-1. NLRP12 can also inhibit non-canonical NF-κB signaling through regulation of TRAF3 and NF-κB inducing kinase (NIK).

Mechanistically, NLRP12 was shown to suppress both canonical and non-canonical NF-κB signaling pathways. The canonical pathway is mediated by translocation of the NF-κB RelA/p50 subunits to the nucleus and is activated in response to TNFR, IL-1R, or TLR signaling. NLRP12 inhibits hyperphosphorylation of the receptor-associated kinase (IRAK-1) that triggers IκBα degradation and p50 nuclear translocation (134, 137). The non-canonical NF-κB pathway is triggered by signaling through receptors, such as CD40, LTβR, or BAFF-R. The signal activates NF-κB inducing kinase (NIK) and IKKα, which leads to p100 cleavage and nuclear translocation of p52 dimers. In the non-canonical NF-κB signaling pathway, NLRP12 interacts with TNF receptor-associated factor (TRAF3) and NIK, which leads to the degradation of NIK and subsequent reduction of p100 cleavage to p52 (Figure 6) (125, 138). ATP binding to NLRP12 is crucial for its inhibitory function, as the cells with an NBD mutant form of NLRP12 are not able to inhibit NF-κB activation. As a result, they produce high levels of proinflammatory cytokines and chemokines (139).

Apart from its inhibitory role in monocytes/macrophages, NLRP12 was shown to enhance the migration of DCs to the draining lymph nodes (140). In the absence of NLRP12, the migration of DCs to the local lymph nodes is significantly decreased, while their abilities for antigen presentation, inflammasome activation, or production of inflammatory cytokines are not impaired. Therefore, *Nlrp12^−/−^* mice fail to generate robust hypersensitivity response against the topical application of hapten-like oxazolone (140).

Consistent with the regulatory function for NLRP12 in innate immune cells, *Nlrp12^−/−^* mice were shown to be highly susceptible to inflammatory diseases of intestine, such as experimental colitis and colon cancer (137, 138). This is due to the increased activation of NF-κB in macrophages of *Nlrp12^−/−^* mice, which results in the production of proinflammatory cytokines and mediators. Consistent with these findings, our work demonstrated a protective role of NLRP12 during CNS inflammation. We showed that lack of NLRP12 potentiated the course of EAE (126). Indeed, *Nlrp12^−^*^/^*^−^* mice developed earlier and more severe EAE compared to the WT mice (126). *In vitro* experiments also confirmed the inhibitory role of NLRP12 in microglia activation and the production of proinflammatory mediators, such as iNOS expression, NO, TNF-α, and IL-6 (126).

Alternatively, a study by Lukens et al. proposed that NLRP12 provokes CNS inflammation in EAE that is related to its regulatory function in T cells (128). Early reports mainly described NLRP12 expression and its anti-inflammatory function in innate immune cells with myeloid origin, such as DC and macrophages (137, 140). Recently, Lukens et al. reported a T cell-intrinsic role for NLRP12, which negatively regulates NF-κB signaling, T cells proliferation, and the secretion of T_H_1/T_H_2/T_H_17 cytokines (128). Therefore, *Nlrp12^−/−^* T cells developed enhanced inflammatory symptoms in T-cell-mediated autoimmune diseases such as colitis and atopic dermatitis (138, 140). These findings support our observation that lack of NLRP12 was associated with increased neuroinflammation and severe scores of EAE (126). Lukens’ study shows that the absence of NLRP12 promoted IL-4 secretion resulting in the development of atypical EAE disease symptoms, including ataxia and impaired balance control (128).

NLRP12 also plays a positive role in T_H_1/T_H_17 differentiation. Using an *in vitro* T-cell differentiation assay, Cai et al. reported a reduced T_H_1 and T_H_17 differentiation in *Nlrp12^−/−^* T cells, while Th2 differentiation remained similar to WT T cells (141). In response to *Brucella abortus in vivo*, Silveira et al. showed that *Nlrp12^−/−^* T cells produced more IFN-γ compared to WT controls. The study did not investigate whether the increased production of IFN-γ was due to high numbers of T_H_1 cells; however, they demonstrated that NLRP12 negatively regulated IL-12 production by macrophages following *B. abortus* infection, which skewed T cell differentiation to T_H_1 (129). Further research is needed to understand how NLRP12 regulates T cell activation and differentiation.

Regarding the dual role of NLRP12 in the regulation of inflammation (142), the inconsistency of results across laboratories might be related to the different environmental conditions that result in different microbiomes, and different knockout strategies to delete NLRP12 gene, which may produce uncontrolled variable phenotypes. Interestingly, two recent studies may provide clues to explain some of the inconsistencies in the NLRP12 literature. In one study, it was shown that some C57BL/6 colonies have acquired a missense mutation in the *Nlrp12* gene and that this can affect neutrophil responses (143). In the second study, genetic ablation of NLRP12 was found to cause significant changes in microbiota landscape in mice (144). Interestingly, they found that cohousing *Nlrp12^−^*^/^*^−^* mice with WT mice attenuates intestinal inflammatory disease in NLRP12-deficient mice. Collectively, these two studies demonstrate that it is important when evaluating a role for NLRP12 in disease to take into consideration differences in microbiota composition in *Nlrp12^−^*^/^*^−^* mice colonies and choice of C57BL/6 wild-type controls. Going forward it will be particularly interesting to determine how modulation of intestinal microflora landscape in NLRP12-deficient mice influences inflammatory responses and disease progression in other models of disease.

### NLRX1

NLRX1 is a recently characterized member of the NLR family that is uniquely localized in the mitochondria (145). The protein is expressed widely in all tissues with the highest expression in heart and muscle (146). Initial studies showed that NLRX1 was located in the outer membrane of mitochondria (145). However, later studies demonstrated that NLRX1 is predominantly located in the matrix of mitochondria (14, 147). The localization of NLRX1 in mitochondria is due to the presence of a functional N-terminal mitochondrial-localization sequence (14). In mitochondria, NLRX1 interacts with UQCRC2, a part of the ubiquinol-cytochrome c reductase complex, that is a part of the mitochondrial respiratory chain (MRC). The MRC generates an electrochemical signal that drives ATP production (148). It also produces ROS in eukaryotic cells that could cause oxidative stress and tissue damage (149). A study by Tattoli et al. showed that NLRX1 induces ROS production in cells treated with TNF-α and double-stranded RNA, which results in increased activation of inflammatory pathways, such as NF-κB (146). Alternatively, Xia et al. reported that NLRX1 acts as a negative regulator of TLR-mediated NF-κB signaling. In resting cells, NLRX1 interacts with TRAF6. However, after cell stimulation with LPS, NLRX1 rapidly dissociates from TRAF6 and binds to the IKK complex, leading to inhibition of IKKα/β phosphorylation and NF-κB activation (Figure 6) (150). Therefore, depending on the experimental conditions, NLRX1 can either activate or inhibit NF-κB signaling.

In the context of viral infections, NLRX1 acts as a negative regulator of the antiviral signaling pathway (145, 151). Once cells are infected with a virus, viral PAMPs are detected by the cytoplasmic RLRs and MDA5, which activate MAVS signaling pathway, resulting in the activation of IRF3, NF-κB, and transcription of type-1 interferon (IFN-1) (145, 151). Moore et al. reported that interaction between NLRX1 and MAVS prevents RIG-I from binding to MAVS, which results in inhibition of NF-κB activation and IFN-1 production (145). Consistent with this finding, Allen et al. showed that embryonic fibroblasts from *Nlrx1^−^*^/^*^−^* mice had increased production of type 1 IFN after viral infection as compared to WT controls (151). In contrast, Rebsamen et al. show NLRX1 has no effect on antiviral response. They reported that NLRX1*^−^*^/^*^−^* embryonic fibroblasts had normal cytokine production in response to Sendai virus infection (152). This finding is in agreement with Soares et al. study, which showed that antiviral signaling pathway is intact in *Nlrx1^−^*^/^*^−^* mice during both *in vivo* and *ex vivo* viral infections (153). Apart from its controversial role in antiviral immune response, NLRX1 can induce autophagy that deletes the cytosolic viral RNA and consequently results in the inhibition of type 1 IFN production. Lei et al. proposed that NLRX1 forms a multimeric complex with the cytosolic autophagy-related (ATG) proteins and a mitochondrial matrix protein, mitochondrial Tu translation elongation factor (TUFM), which is known to initiate autophagic responses (154). For this reason, NLRX1 deficient cells enhance type I IFN production and are more efficient to restrict the replication of a variety of viruses compared to WT cells.

Studies summarized here highlight the regulatory role of NLRX1 in the immune responses against viruses. However, emerging studies have also identified key roles for NLRX1 in multiple autoinflammatory and autoimmune disease models. For instance, a recent work by Eitas et al. demonstrated a protective role of NLRX1 in EAE by suppressing macrophage/microglial activation (155). In this study, *Nlrx1^−/−^* mice showed increased cytokine production and enhanced tissue damage during EAE compared to the WT mice (155). This finding is consistent with a recent study by Allen et al., which also showed the anti-inflammatory function of NLRX1 in sterile CNS inflammation, such as traumatic brain injury (156). Mechanical trauma to the CNS results in the disruption of the cellular microenvironment leading to massive necrotic and apoptotic loss of neuronal and glia populations. *Nlrx1^−/−^* mice exhibited significantly larger brain lesions and increased motor deficits following brain injury. Their data indicates that NLRX1 attenuates NF-κB signaling and IL-6 production in microglia (157).

It is also reported that NLRX1 regulates mitochondrial dynamics and cell death. Recently, our research group showed that NLRX1 protects the neuronal-like cell line (N2A cells) from necrosis (158). We found an increased number of mitochondria in NLRX1-overexpressed N2A cells compared to control cells, which was associated with increased phosphorylation of DRP1 and mitochondrial fission. As a result, NLRX1 switched the cell death from necrosis toward apoptosis, which inhibits neurodegeneration by preventing the release of inflammatory mediators in the tissue environment and maintaining the tissue homeostasis (158). Consistent with our observations, a study by Girardin’s group showed that NLRX1 accelerates intrinsic apoptotic pathway induced by prolonged cellular stress or glucose starvation (159).

Interestingly, recent studies suggest that in addition to playing prominent roles in the regulation of innate immune cells, NLRX1 can also affect adaptive immunity by inhibiting T cell proliferation and differentiation (160). In dextran sodium sulfate-induced colitis mouse model, lack of NLRX1 results in enhanced T_H_1- and T_H_17-related inflammatory cytokines, such as IFN-γ, TNF-α, and IL-17, and consequently increased the severity of the disease (160). *In vitro* experiments revealed that *Nlrx1^−/−^* T cells have a greater ability to proliferate and differentiate into T_H_17 cells. The T-cell intrinsic role of NLRX1 was confirmed in adoptive-transfer model of colitis. The *Rag^−/−^* mice receiving *Nlrx1^−/−^* T cells experienced more severe clinical disease and increased numbers of T_H_1 and T_H_17 cells in spleen and colonic lamina propria (160).

### NLRC3

NLRC3 is predominantly expressed in cells of the immune system, particularly in T cells (134). NLRC3 functions as a novel suppressor of T cell activation. It inhibits NF-κB, AP-1, and NFAT transcriptional activation in Jurkat T cells downstream of CD3/CD28 stimulation or treatment with PMA/ionomycin (134). Studies of *Nlrc3*^−/−^ mice confirmed the inhibition of proinflammatory signaling, K63-linked ubiquitination of TRAF6, and nuclear translocation of NF-κB by NLRC3 (161). Interestingly, it was shown that *nlrc3-like* gene is required for microglia development in Zebrafish (162). In *nlrc3-like* mutants, primitive macrophages gain an inflammatory phenotype with increased proinflammatory cytokines that prevent their migration into the brain and subsequent differentiation into microglia. This study suggests that *nlrc3-like* serves as a critical regulator of microglia development in Zebrafish; however, future studies in vertebrate models are needed to fully elucidate roles for NLRC3 in neuroinflammatory diseases.

### NLRs in Other Neuroinflammatory Diseases

The importance of NLR proteins can be further appreciated by their crucial role in inflammatory diseases where a simple mutation in these genes can result in pathology (163) Cryopyrin-Associated Periodic Syndromes (CAPS) are a group of autoinflammatory syndromes resulting from an autosomal dominant mutation in the *Nlrp3* gene (164). Variants of NLRP1 proteins have been shown to be associated with vitiligo, an autoimmune disease resulting in areas of skin hypopigmentation as a consequence of melanocytes damage (91). Moreover, mutations in the NBD of *Nod2* gene result in Blau syndrome, an autosomal dominant disorder that is characterized by skin rashes, arthritis, and granulomatous uveitis (165). The proinflammatory NLRP3 contributes to the pathology of a broad spectrum of neurological diseases, such as stroke (166), traumatic injury (167), and neurodegenerative diseases, including Alzheimer (168) and amyotrophic lateral sclerosis (ALS) (169).

## Therapeutic Potential of Targeting NLRs in Disease

### Inhibition of Inflammatory NLRs

Targeting NLR-mediated inflammasome activation is an attractive therapeutic approach that is actively being investigated to treat a multitude of autoinflammatory and autoimmune disorders. As described above in greater detail, multiple NLRs are known to coordinate inflammasome-mediated production of IL-1β and IL-18, as well as a caspase-1-dependent form of cell death known as pyroptosis. Therapeutic molecules targeting IL-1 are being used in the clinic for many years. Currently, there are three approved IL-1 blockers, including the IL-1 receptor antagonist, anakinra; a soluble decoy receptor, rilonacept; and a neutralizing monoclonal anti-IL-1β antibody, canakinumab (170). There are no approved treatments to block IL-18 in humans at this time, however, a recombinant human IL-18 binding protein (Tadekinig alfa) is currently in clinical trials (171). Despite their notable efficacy, anticytokine drugs are not able to inhibit other inflammasome-associated pathologies, such as caspase-1-mediated pyroptosis. Therefore, therapeutics that directly target inflammasome activation may offer greater efficacy over strategies that only target inflammasome-derived cytokines. In this respect, two caspase-1 inhibitors have been recently developed and tested in clinical trials. These are orally absorbed prodrugs: Pralnacasan (VX-740) and Belnacasan (VX-765), that selectively inhibit the activity of caspase-1 (172).

IFN-β is one of the most widely prescribed disease modifying therapies for relapsing-remitting MS. IFN-β exerts its anti-inflammatory effect through the suppression of NLRP1 and NLRP3 inflammasome and IL-1β production, as it was shown in mouse bone marrow-derived macrophages and blood monocytes isolated from IFN-β treated MS patients (173). The NLRP3 inflammasome is associated with the response to IFN-β in patients with MS (112), which indicates that IFN-β specifically inhibits NLRP3 inflammasome.

There are two small-molecule inhibitors of inflammasome that specifically inhibit NLRP3 inflammasome. MCC950 is a diarylsulfonylurea-containing compound that blocks NLRP3-induced ASC oligomerization in mouse and human macrophages (174). MCC950 acts specifically on the NLRP3 inflammasome and does not inhibit the activation of NLRP1, AIM2, or NLRC4 inflammasomes (174). It was shown that treatment of mice with MCC950 delayed the onset and reduced the severity of EAE (174). The ketone metabolite β-hydroxybutyrate (BHB) is another small-molecule inhibitor of inflammasome that specifically inhibits NLRP3-induced ASC oligomerization (175).

Another approach for inhibiting inflammasomes is using MicroRNAs, the single-stranded non-coding RNA molecules that bind to the 3-untranslated region of mRNAs to regulate gene expression (176). MicroRNA-223 binds to a conserved site in the 3 UTR of the NLRP3 transcript, suppressing the protein expression, thus, inhibiting NLRP3 inflammasome (177). The therapeutic application of MicroRNA-223 is currently under investigation in animal models (178). Despite the availability of miRNA therapeutics in human clinical trials, none of them are currently known to target inflammasome signaling (179).

Previous studies suggest that mitochondrial dysfunction plays a crucial role in the pathogenesis of neurodegenerative disorders, such as Parkinson’s disease (PD), Alzheimer’s disease (AD), and MS (180, 181). Mitochondrial dysfunction generates ROS, which triggers NLRP3 oligomerization and activates inflammasome (182). Therefore, the inhibition of mitochondrial ROS (mtROS) using mitochondria-targeted antioxidants is another approach to suppress inflammasome (183). A recent publication shows that MitoQ, a mitochondria-targeted antioxidant derived from ubiquinone, attenuates experimental mouse colitis by inhibiting the NLRP3 inflammasome and the production of inflammatory cytokines (184). The neuroprotective effects of MitoQ have been confirmed in EAE mice, in which treatment with MitoQ reduced axonal inflammation and neurological disabilities (185). These findings suggest that novel mitochondria-targeted antioxidants could be promising therapeutic targets for MS treatment (186).

### Stimulation of Anti-inflammatory NLRs

NF-κB is a master regulator inflammation that plays pivotal roles in the transcriptional control of a vast majority of inflammatory mediators, including TNF-α, IL-6, pro-IL-1β, pro-IL-18, procaspase-1, and NLRP3 (187). In addition to coordinating the expression of many inflammasome-related molecules, NF-κB can also potently affect inflammatory responses through its regulation of chemokine and adhesion molecule production, and its control of cell proliferation and differentiation (188). Therefore, the activation of NF-κB pathway is not only required for the assembly of inflammasome complex but required for the activation and recruitment of inflammatory cells to the site of inflammation. The contribution of NF-κB in such a broad spectrum of inflammatory responses has spurred great interest in the development of NF-κB inhibitors to treat MS.

Multiple studies have demonstrated that NF-κB inhibition, both in peripheral immune cells and in the CNS, is protective in EAE, suggesting that pharmacological targeting of the NF-κB pathway might have a therapeutic effect in MS. A number of currently prescribed MS drugs, including fingolimod, teriflunomide, and dimethyl fumarate, have been reported to indirectly modulate NF-κB signaling (189). There are several NF-κB specific inhibitors, such as DHMEQ and bindarit, which prevent the nuclear translocation of the p65 (190) or reduce the phosphorylation of IκBα and p65 (191). NF-κB specific inhibitors showed potent anti-inflammatory and anticancer activities in many animal models (192). However, their anti-inflammatory activity in autoimmune diseases requires further investigation.

During the proinflammatory response, anti-inflammatory NLRs provide simultaneous and opposing down-regulation of inflammation that target not only immune cells and their mediators but also CNS-resident cells. Therefore, targeted approaches to boost the expression or function of anti-inflammatory NLRs would serve as a novel strategy to treat neuroinflammatory disease. Anti-inflammatory NLRs, such as NLRX1 and NLRP12, are the natural inhibitors of NF-κB that proficiently switch off the inflammatory cascade upstream of NF-κB signaling. Ligands for NLRX1 and NLRP12 have remained poorly described. However, a number of NLRX1 binding molecules and inhibitors were recently identified using a molecular docking approach to screen natural products and lipid databases (193). This study by Lu et al. revealed that punicic acid (PUA), eleostearic acid (ESA), and docosahexaenoic acid (DHA) can bind to the C-terminal fragment of the human NLRX1. Using Nlrx1*^−^*^/^*^−^* cells, the study showed that PUA and DHA suppressed the NF-κB activity in macrophages in a NLRX1-dependent mechanism *in vitro*. The NLRX1-dependent mechanism of PUA was further confirmed in the DSS model of colitis. In these studies, DSS-challenged mice were treated orally with either PUA (40 mg/kg body) or PBS. The WT mice treated with PUA showed significantly lower TNF-α and ameliorated mucosal inflammation compared to *Nlrx1^−/−^* counterparts (193). This study shows a great potential of NLRX1 in the treatment of inflammatory diseases.

Pidotimod (3-L-pyroglutamyl-L-thiazolidine-4-carboxylic acid) is a synthetic dipeptide immunomodulator that is largely used for treatment of respiratory tract infections, asthma, and chronic obstructive pulmonary disease (194). Previous studies show that pidotimod acts as an immunostimulant that induces DC maturation and T cell differentiation toward a T_H_1 phenotype (195–197). A recent publication by Fogli et al. demonstrated the anti-inflammatory property of pidotimod in TLR-stimulated macrophages, which was associated with the increased expression of NLRP12 at both levels of mRNA and protein (198). Silencing NLRP12 expression recovered the proinflammatory response of pidotimod-treated cells, which suggests that the anti-inflammatory response of pidotimod was related to the levels of NLRP12 expression (198). These findings pave the way for the development of innovative treatments for inflammatory diseases through activating anti-inflammatory NLRs that naturally control the inflammatory pathways within cells.

## Perspectives

Out of the 23 known NLRs in humans, only a handful of NLRs have been formally studied in MS to date. Given the prominent role of NLRs in host-pathogen interactions and inflammatory conditions, we anticipate that additional NLR signaling pathways will be found to impact neuroinflammatory diseases in the coming years. There are numerous NLRs that have been recently identified to affect inflammatory responses in other disease models [e.g., NLRP1, NLRP6, NLRC3, and NLRP4 (163, 199)] and we believe that it is only a matter of time until we come to fully appreciate the roles of these proteins in MS.

In recent years, there has been tremendous interest in the role that B cells play in MS due to the recent successes of anti-CD20-mediated B cell depletion in the treatment of both relapsing and primary progressive MS (200). B lymphocyte differentiation into plasma cells results in the secretion of immunoglobulins, which can bind and activate complement or induce antibody-dependent cytotoxicity (31). Surprisingly, little is currently known about NLR-dependent control of B cell responses in the context of demyelinating neuroinflammatory disease. Therefore, given the potent effect of NLRs on the homeostasis of the immune system, we expect that in the next few years MS research will focus on the role of NLRs in B cells.

Moreover, we may speculate that NLRs will emerge as attractive targets for therapeutic intervention in multiple neurological disorders, including MS, PD, AD, traumatic spinal cord, brain injury, and stroke.

## Author Contributions

MG and DG designed the format and figures and wrote the entire manuscript. TM contributed to the writing some sections of the manuscript including NLRP12 and innate immunity in the CNS. KG, AA, and JL contributed to the conceptual reading and critical editing of the manuscript. All authors read and approved the manuscript.

## Conflict of Interest Statement

The authors declare that the research was conducted in the absence of any commercial or financial relationships that could be construed as a potential conflict of interest.
