# Erratum: NLR-Dependent Regulation of Inflammation in Multiple Sclerosis

**DOI:** 10.3389/fimmu.2018.00404

**Published:** 2018-02-28

**Authors:** Frontiers Production Office

**Affiliations:** ^1^Frontiers Media SA, Lausanne, Switzerland

**Keywords:** NLRs, multiple sclerosis, NLRP3, NLRP12, NLRX1, inflammation, astrocytes, microglia

Due to a typesetting error, the title was given incorrectly as “NLR-Dependent Regulation of Inflammation in Multiple Sclerosis- Dependent Regulation of Inflammation in Multiple Sclerosis.” The correct title is “NLR-Dependent Regulation of Inflammation in Multiple Sclerosis.”

The publisher apologizes for this error and the original article has been updated to reflect this. This error does not change the scientific conclusions of the article in any way.

